# Immunotherapy yields breed-specific worst survival outcomes among three investigated therapies in French bulldogs with high-grade glioma

**DOI:** 10.3389/fvets.2025.1532439

**Published:** 2025-03-19

**Authors:** Susan A. Arnold, Amanda R. Taylor, Katherine Hansen, Vijay Agarwal, Walter C. Low, G. Elizabeth Pluhar

**Affiliations:** ^1^Department of Veterinary Clinical Sciences, University of Minnesota College of Veterinary Medicine, Saint Paul, MN, United States; ^2^Southeast Veterinary Neurology, Virginia Beach, VA, United States; ^3^VCA Bay Area Veterinary Specialists, San Leandro, CA, United States; ^4^Department of Neurosurgery, Albert Einstein College of Medicine, Bronx, NY, United States; ^5^Department of Neurosurgery, University of Minnesota Medical School, Minneapolis, MN, United States

**Keywords:** French bulldog, high-grade glioma, oligodendroglioma, immunotherapy, radiation therapy, sonodynamic therapy

## Abstract

**Introduction:**

French bulldogs are one of the most popular dog breeds in the United States and are also among breeds with the highest risk for developing high-grade glioma (HGG). With limited treatment options and high translational value for studying canine HGG to advance understanding of human glioblastoma (GB), a variety of novel treatment options have been investigated. In other forms of cancer, immunotherapy has shown promising results, garnering interest in the treatment of HGG. Yet, when an immunotherapy-based clinical trial was conducted, a marked survival disparity in French bulldog patients compared to other breeds was observed.

**Methods:**

This retrospective, multi-institutional study was conducted to examine survival outcomes in immunotherapy-treated French bulldogs compared to closely related breeds, and to French bulldogs treated with several other treatment modalities.

**Results:**

French bulldogs treated with immunotherapy experienced significantly shorter overall survival (OS) than boxers and Boston terriers (132 vs. 221 days, respectively). French bulldogs treated with immunotherapy had no significant difference in OS compared to French bulldogs treated palliatively, whereas dogs treated with either a novel therapy involving sonodynamic therapy or stereotactic radiation therapy had significantly longer OS.

**Discussion:**

This study provides evidence for an immunotherapy-resistant form of HGG in French bulldogs, suggesting that the breed harbors key molecular differences affecting the tumor and tumor-immune microenvironment and subsequent poor response to treatment.

## 1 Introduction

The French bulldog became the most popular dog breed in the United States in 2022. Formerly a relatively uncommon breed, its popularity rose exponentially from 2010 to the present day. Numerous factors contributed to its succession, including its appeal as an apartment dog, baby-like face, and popularity among celebrities and social media influencers (1). As has occurred for other exponentially popular dog breeds in the past, the public demand for French bulldogs has led to the recognition of serious breed-associated health concerns (2). Between reproductive, respiratory, dermatologic, cardiac, and neurologic issues, French bulldogs have overall poorer health than other breeds due to their inherited, extreme conformational abnormalities (3).

French bulldogs belong to the phylogenetic clade that contains the breeds most often affected by high-grade oligodendroglioma (HGO), a subset of high-grade glioma (HGG) (4). This is an aggressive, primary brain tumor associated with poor survival. When compared to other dog breeds, French bulldogs, boxers, and Boston terriers have a markedly higher risk of HGO (5).

Current treatment options for brain lesions that are suspected to be canine HGO based on MRI appearance are limited in efficacy and availability. Interpreting survival data is difficult, since tolerance for clinical signs and threshold for euthanasia criteria are highly variable among owners of dogs with HGO, and often a definitive histopathologic diagnosis is not made (6). When treated with palliative care, the reported median survival time for HGG is 38 days (range 1–492 days) (6). Surgical resection alone does not improve survival outcomes over palliative care alone (7). Radiation therapy can result in average survival times ranging from approximately 300 to 700 days, but requires multiple anesthetic procedures, which is a challenge for brachycephalic patients such as French bulldogs prone to anesthesia-related regurgitation and is cost prohibitive for many pet owners (8–12). Thus, our group investigates novel therapies for canine HGG to improve patient outcomes. Another impetus is to inform human glioblastoma (GB) clinical trials, for which the canine HGO is a strong, spontaneous model (13–15).

Our clinical trials have mainly focused on providing immunotherapy-based treatments following surgical resection of HGG, the majority of which are HGO. Following maximal, safe surgical resection, all trial patients receive one of the various combinations of immunotherapy. Briefly, the immunotherapy treatments we have investigated include administration of the co-stimulatory molecule OX40L, Flt3L and thymidine kinase gene therapy, autologous tumor lysate therapy, and inhibition of the CD200 immune checkpoint.

Throughout enrollment, a trend of poor outcome in French bulldogs compared to other breeds was observed. The objective of this study was to perform a retrospective evaluation of patient records to determine whether French bulldogs treated with immunotherapy for their HGO had worse survival outcomes compared to other breeds. To determine whether our observation of poor survival in French bulldogs with HGO extended beyond immunotherapy, we compared the survival of immunotherapy-treated French bulldogs with French bulldogs treated either palliatively, in an unrelated clinical trial that did not include primary immunotherapy, or with stereotactic radiation therapy.

## 2 Materials and methods

### 2.1 Immunotherapy patients

Clinical records were searched for all dogs that had undergone treatment for HGO as part of one of our clinical trials between 2012 and 2023. The clinical trial protocols were approved by the University of Minnesota Institutional Animal Care and Use Committee (IACUC). All dogs in these studies were first presumptively diagnosed via magnetic resonance imaging (MRI) with a solitary intraparenchymal prosencephalic tumor with features consistent with a high-grade glioma (HGG) (6). All dogs underwent maximal, safe surgical resection and immediate post-operative MRIs to assess extent of resection. Following recovery from anesthesia, all dogs recovered in our intensive care unit for at least 24 h. All dogs with a history of seizures continued to receive their chronic anticonvulsants as previously prescribed. All dogs were treated with a tapering course of corticosteroids over 10–14 days. Histopathology was performed on intraoperative tumor samples, and all dogs were confirmed to have HGO using the updated diagnostic classification of canine glioma (16). Details regarding the treatment protocols for each immunotherapy trial are available in Supplementary material.

For inclusion, a case had to have breed, age, sex, histopathological confirmation of HGG, tumor location, and PFS and OS information available. Tumor locations were classified as frontal, parietal, occipital, and temporal (including piriform lobe). French bulldogs, boxers, and Boston terriers were included. Boxers and Boston terriers were chosen as the comparison group because they belong to the same phylogenetic clade as French bulldogs and have similarly high predisposition for HGG. Yet, poor treatment response was not observed in boxers and Boston terriers in response to immunotherapy.

In total, there were six types of immunotherapy that were included in this study, created from various combinations of autologous tumor lysate therapy, immune checkpoint inhibition against the CD200 immune checkpoint, gene therapy and temozolomide.

### 2.2 Non-immunotherapy patients

To investigate whether the poor treatment response in French bulldogs was limited to immunotherapy, three additional patient groups were included: French bulldogs with prosencephalic tumors suspected to be HGG treated palliatively, French bulldogs with prosencephalic tumors treated in another trial that did not involve immunotherapy, and French bulldogs with prosencephalic tumors suspected to be HGG treated with stereotactic radiation therapy.

For the dogs treated palliatively, clinical records of all four locations of Southeast Veterinary Neurology (SEVN) were searched for all French bulldogs that had been presumptively diagnosed via MRI with high-grade glioma between 2012 and 2024. Palliative care consisted of anticonvulsants for dogs with a history of seizures secondary to their glioma and anti-inflammatory doses of corticosteroids. For inclusion in the study, the same data as detailed above were required, other than histopathological confirmation of HGO. Dogs that were euthanized within 24 h of their MRI diagnosis were excluded.

The non-immunotherapy clinical trial consisted of the same surgical protocol as the immunotherapy trial, along with sonodynamic therapy (SDT) consisting of administration of 5-aminolevulinic acid (5-ALA) and whole-brain low-intensity diffuse ultrasound every 21–28 days. The same data as detailed for the immunotherapy patients were required.

For the dogs treated with stereotactic radiation therapy, clinical records of the University of California at Davis School of Veterinary Medicine Small Animal Clinic were searched for all French bulldogs that had been treated with stereotactic radiation therapy (SRT) for presumptive high-grade glioma between 2012 and 2024. SRT consisted of three consecutive days of 8 Gray (Gy) fractions delivered by intensity-modulated or volumetric-acr radiotherapy with a Varian TrueBeam, except for one case where the dose was delivered with a Clinac linear accelerator using the BrainLab cone system as described elsewhere (17). For inclusion in the study, the same data as detailed above for dogs treated palliatively were required.

### 2.3 Survival evaluation criteria

When possible, both progression-free survival (PFS) and overall survival (OS) were recorded, with the date of MRI diagnosis for all treatment groups serving as the reference point. Data were censored if dogs received additional treatments based on the development of tumor regrowth or if they were lost to follow up. Both the mean and median OS for each group were determined. We included both forms of OS reporting because median OS is insensitive to outliers, particularly long-term survivors (18, 19). When evaluating the treatment groups, ensuring that long-term survivors were also accounted for was important in describing the various possible outcomes for a given therapy, particularly given the heterogeneity of glioma (20, 21).

### 2.4 Statistical analyses

Differences in patient details between immunotherapy-treated French bulldogs, boxers and Boston terriers were examined. Chi-squared tests were used to examine differences in patient sex and tumor location. After confirming that age at diagnosis was normally distributed, a Welch’s two sample t-test was used to examine differences in age at diagnosis between breeds. Within the analysis of French bulldogs treated with four different forms of therapy, Chi-squared tests were again used to examine differences in patient sex and tumor location. After confirming normality, a one-way analysis of variance (ANOVA) was used to examine differences in age at diagnosis between treatment groups. Tukey’s *post hoc* testing was done to examine significant differences observed in the ANOVA.

To evaluate survival outcomes for immunotherapy-treated French bulldogs compared to other breeds, a Kaplan–Meier curve was created. A Cox proportional hazards model for multivariate analysis was created to assess the effects of breed (French bulldogs versus boxers and Boston terriers), age, sex, and treatment hazard rates on OS. Tumor location was excluded from the model because there was a high correlation between parietal location of tumors and the French bulldog breed.

To evaluate survival outcomes for immunotherapy-treated French bulldogs compared to French bulldogs treated with either palliative care, 5-ALA and SDT, or SRT, a Kaplan–Meier curve was created. A Cox proportional hazards model for multivariate analysis was created to assess the effects of treatment, age, sex, and tumor location hazard rates on OS.

Significance was considered with a cutoff of *p* < 0.05. The statistical software R 4.4.1 (R Foundation for Statistical Computing, Vienna, Austria) was used for all analyses.

## 3 Results

### 3.1 Patient details

A total of 85 dogs were included in this study. In the immunotherapy group, there were seven French bulldogs, 17 boxers, and 10 Boston terriers. The same seven French bulldogs were then used in an evaluation along with 31 French bulldogs treated with palliative care, eight French bulldogs treated with SDT, and 12 French bulldogs treated with SRT. Details about the dogs, including breed age, sex, tumor location, treatment, and overall survival are summarized in Tables 1, 2.

**Table 1:** Patient details for dogs treated with immunotherapy.

Breed	Median age (years)	Sex	Tumor location	Treatment
French bulldog *n* = 7	6.25	MN *n* = 4	Frontal *n* = 2	CD200 + vax *n* = 5
		FS *n* = 3	Parietal *n* = 3	OX40L and vax *n* = 1
			Temporal *n* = 2	OX40L + vax + TMZ *n* = 1
Boxer *n* = 17	8	MN *n* = 10	Frontal *n* = 9	CD200 + vax *n* = 8
		MI *n* = 2	Parietal *n* = 1	OX40L + vax *n* = 4
		FS *n* = 5	Temporal *n* = 7	OX40L + vax + TMZ *n* = 4
				OX40L + TMZ *n* = 2
Boston terrier *n* = 10	8.5	MN *n* = 7	Frontal *n* = 3	CD200 + vax *n* = 6
		FS *n* = 3	Parietal *n* = 2	OX40L + vax *n* = 2
			Temporal *n* = 4	OX40L + vax + TMZ *n* = 2
			Occipital *n* = 1	

**Table 2:** Patient details for French bulldogs treated with immunotherapy, palliative care, sonodynamic therapy, and stereotactic radiation therapy.

Treatment group	Median age (years)	Sex	Tumor location
Immunotherapy *N* = 7	6.25	MN *n* = 4	Frontal *n* = 2
		FS *n* = 3	Parietal *n* = 3
			Temporal *n* = 2
Palliative care *N* = 31	9.7	MN *n* = 13	Frontal *n* = 14
		MI *n* = 3	Parietal *n* = 6
		FS *n* = 13	Temporal *n* = 10
		FI *n* = 2	Occipital *n* = 1
Sonodynamic therapy *N* = 8	6	MN *n* = 3	Frontal *n* = 3
		FS *n* = 5	Parietal *n* = 3
			Temporal *n* = 2
Stereotactic radiation therapy *N* = 12	7.85	MN *n* = 7	Frontal *n* = 6
		FS *n* = 4	Parietal *n* = 1
		MI *n* = 1	Temporal *n* = 5

Between French bulldogs and boxers and Boston terriers treated with immunotherapy, there were no significant differences in sex (X-squared = 0.85, df = 2, *p* = 0.65), tumor location (X-squared = 3.98, df = 3, *p* = 0.27), or age at diagnosis (*t* = 1.38, df = 9.34, *p* = 0.20). Between French bulldogs treated with immunotherapy, palliative care, SDT, and SRT, there were no significant differences in sex (X-squared = 4.94, df = 9, *p* = 0.84), or tumor location (X-squared = 5.22, df = 9, *p* = 0.81). In the ANOVA, age at diagnosis was significantly different between treatment groups (*F* value = 3.10, *p* = 0.034), but in the *post hoc* analysis pairwise comparisons, there were no significant differences in age at diagnosis between treatment groups.

### 3.2 Survival analysis for immunotherapy-treated French bulldogs compared to boxers and Boston terriers

**Figure 1:**
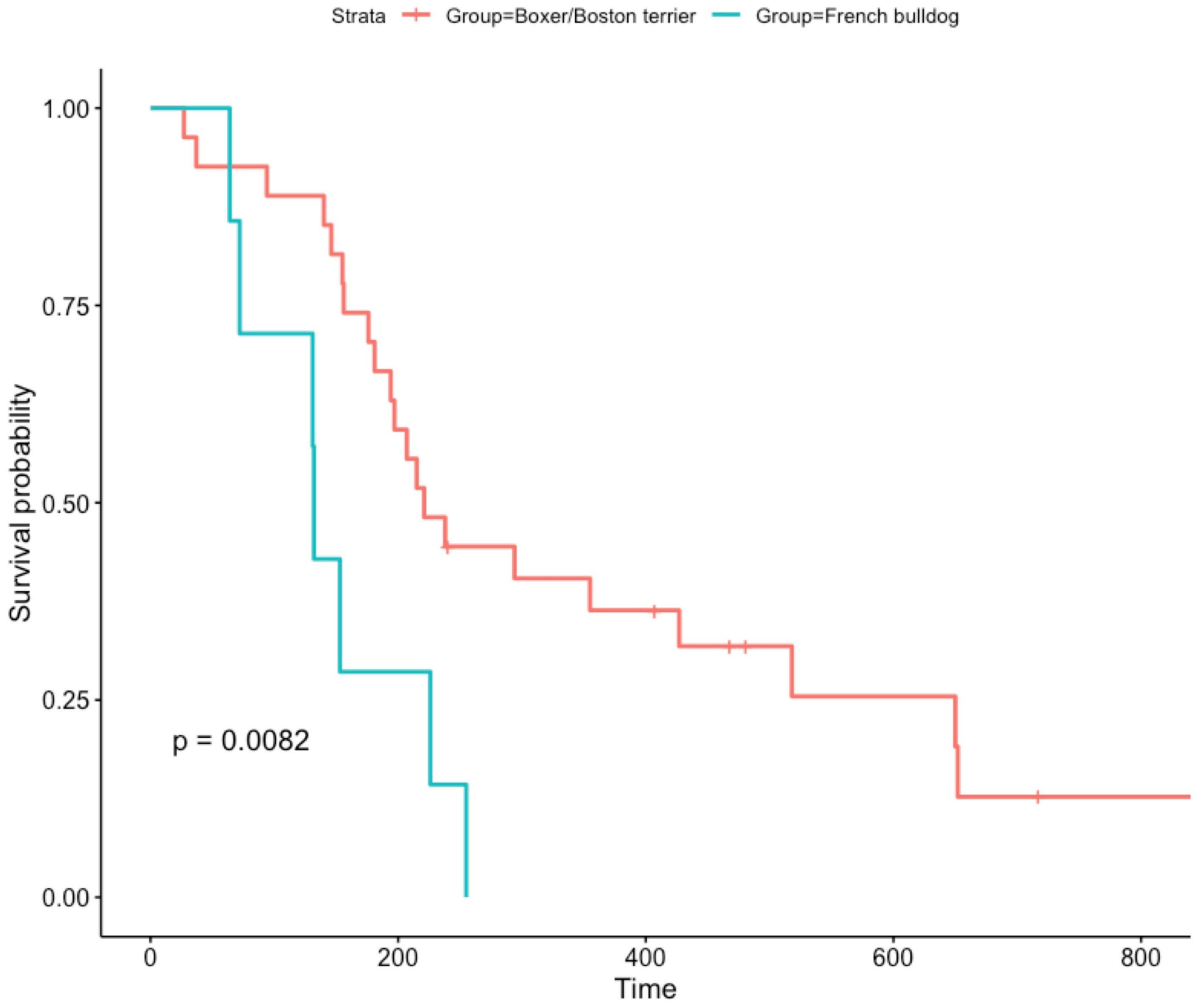
Kaplan–Meier curve showing overall survival for dogs treated with immunotherapy. Censored data are indicated by a tick mark. Note that although there were some boxers and Boston terriers that died shortly after treatment, there were many that had markedly prolonged survival times. Comparatively, nearly all French bulldogs died before 200 days.

PFS was unavailable for the majority of patients, given that few of them underwent serial MRIs to monitor treatment response. When examining OS in dogs treated with immunotherapy, French bulldogs had a significantly shorter OS compared to boxers and Boston terriers (*p* = 0.008) (Figure 1). The median OS for French bulldogs was 132 days (IQR 88), and the mean was 148 days (95% CI 94.4–200.8). The median OS for boxers and Boston terriers was 221 days (IQR 218), and the mean OS was 314 days (95% CI 231.1–396.0).

**Figure 2:**
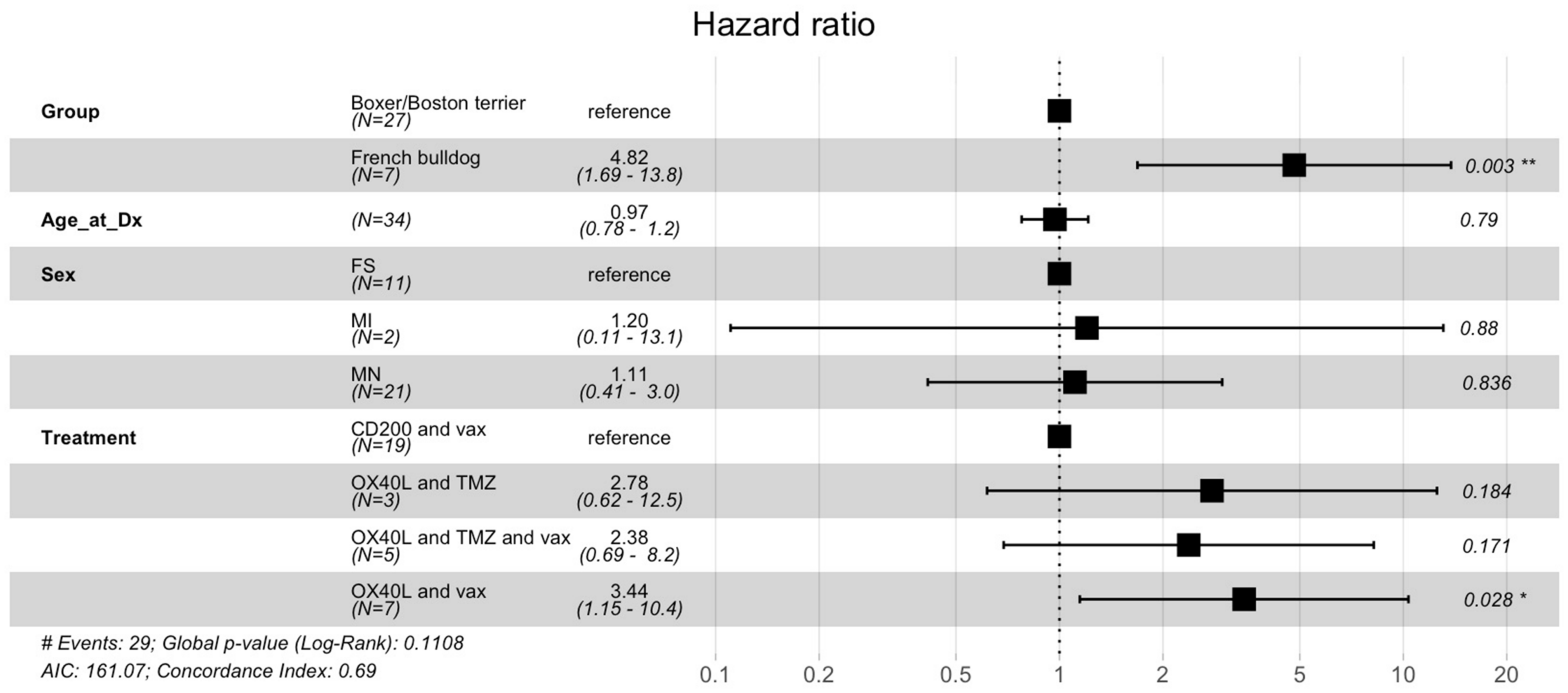
Cox proportional hazards ratio model for overall survival for dogs treated with immunotherapy. Of the various components included in this analysis, breed had the largest and most statistically significant hazard ratio.

The Cox proportional hazards model to examine the association between breed, age, sex, immunotherapy treatment type and overall survival showed that French bulldog breed status was significantly associated with a higher risk of death (hazard ratio = 5.09, 95% confidence interval: 1.61–16.2, *p* = 0.006) (Figure 2). Additionally, treatment with OX40L and vaccines was significantly associated with a higher risk of death (HR = 3.44, 95% CI: 1.15–10.4, *p* = 0.028).

### 3.3 Survival analysis for French bulldogs treated with immunotherapy compared to palliative care, sonodynamic therapy, and stereotactic radiation therapy

**Figure 3:**
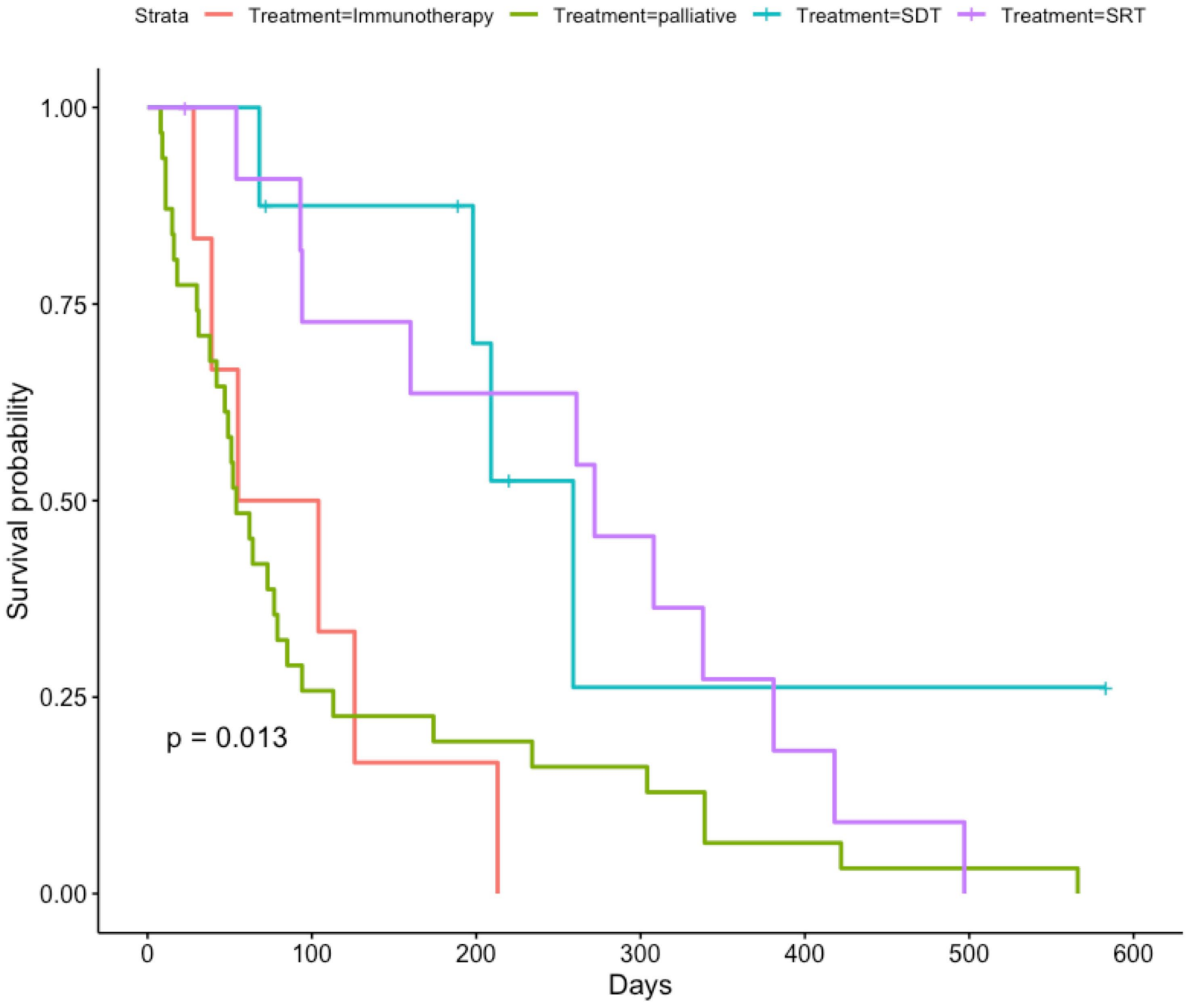
Kaplan–Meier curve showing overall survival for French bulldogs treated with immunotherapy, palliative care, sonodynamic therapy, and stereotactic radiation therapy. Censored data are indicated by a tick mark. Note that the French bulldogs treated with immunotherapy most closely mirrored the French bulldogs treated with palliative care. Notably, though, there were many dogs treated with palliative care that experienced prolonged survival times, whereas nearly none of the dogs treated with immunotherapy survived longer than 200 days.

When examining French bulldogs treated with immunotherapy, palliative care, stereotactic radiation therapy (SRT), and sonodynamic therapy (SDT), there were significant differences in survival among groups (*p* = 0.013) (Figure 3). The median OS for French bulldogs treated with immunotherapy was 132 days (IQR 88), and the mean OS was 148 days (95% CI 94.3–200.8). The median OS for French bulldogs treated with palliative care was 54 days (IQR 73), and the mean OS was 113 days (95% CI 64.1–162.2). The median OS for French bulldogs treated with SDT was 204 days (IQR 70), and the mean OS was 225 days (95% CI 113.7–335.8). The median OS for French bulldogs treated with SRT was 267 days (IQR 255) and the mean OS was 242 days (95% CI 153.9–329.3).

**Figure 4:**
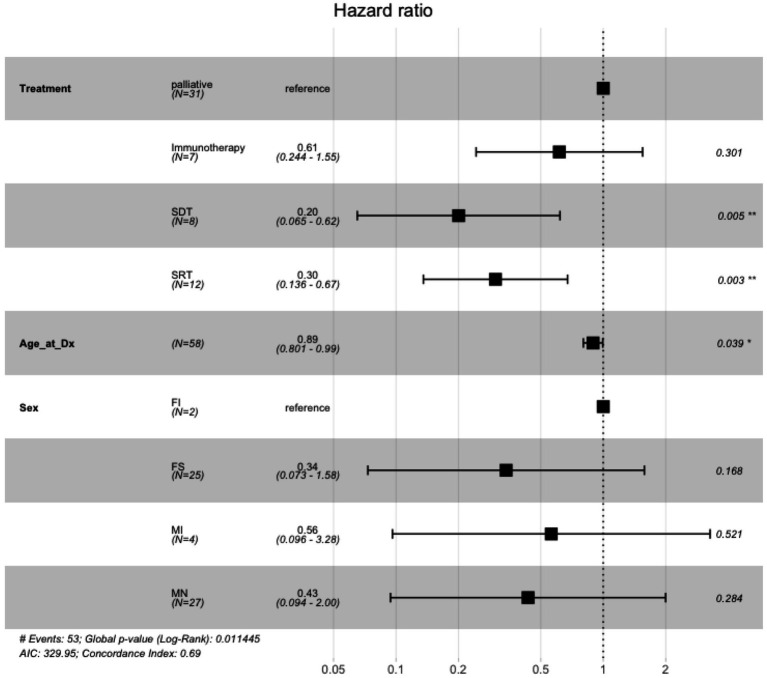
Cox proportional hazards ratio model for overall survival for French bulldogs treated with immunotherapy, palliative care, sonodynamic therapy, and stereotactic radiation therapy. Palliative care was used as the baseline for treatment comparisons. Compared to palliative care, immunotherapy was not associated with a statistically significant difference in hazard ratio, whereas both SDT and SRT were associated with statistically significant differences in hazard ratios.

The Cox proportional hazards model to examine the association between treatment type, age, sex, and overall survival showed that when compared to palliative care, there was no significant difference in risk of death in dogs treated with immunotherapy (hazard ratio = 0.61, 95% confidence interval: 0.24 to 1.55, *p* = 0.37), whereas dogs treated with SDT and SRT had a significantly lower risk of death (SDT hazard ratio 0.20, 95% CI: 0.07 to 0.63, *p* = 0.006; SRT hazard ratio 0.30, 95% CI: 0.14 to 0.68, *p* = 0.004) (Figure 4). Additionally, age at diagnosis was significantly associated with risk of death, such that increased age was associated with lower risk of death (hazard ratio 0.89, 95% CI: 0.80 to 1.00, *p* = 0.041).

## 4 Discussion

The purpose of this study was to characterize the survival outcomes of HGG-bearing French bulldogs. When treated with immunotherapy for high-grade glioma, French bulldogs in this study had a significantly worse overall survival (OS) than boxers and Boston terriers. The French bulldog and Boston terrier are more closely related to each other than either to the boxer, yet Boston terriers did not share the same poor outcome as French bulldogs (4). This provides further evidence that despite their largely similar genetic makeups, there are features that are unique to French bulldogs that predispose them to a worse outcome when treated with immunotherapy for their HGG than other breeds.

When we then evaluated survival outcomes in French bulldogs treated with different types of HGG therapy, we observed that when compared to palliative care, there was no significant difference in survival time of French bulldogs treated with immunotherapy, whereas dogs treated with SDT or SRT survived significantly longer. The median OS observed in the French bulldogs treated with palliative care was 54 days, which is similar to the reported survival time across breeds of 38 days (6). This suggests that HGG in French bulldogs is not inherently more aggressive than HGG of other breeds.

The median OS observed in the French bulldogs treated with SRT was 267 days, which is at the low-end of reported median OS times for all breeds in various studies (226 to 430 days) (8, 22–24). It is also possible that French Bulldogs have poorer outcomes with SRT compared to other breeds, but this hypothesis is outside the scope of the current publication. Several features impact the reliability of comparing SRT survival outcomes across studies. First, SRT protocols differ widely between institutions. Second, reported data are often retrospective, and feature small case numbers. Finally, a diagnosis of glioma is presumed, rather than histologically confirmed, which means that glioma mimics, such as ischemic strokes, inflammatory lesions or granulomas, may be erroneously included, and outcomes are not delineated between low and high-grade tumors or astrocytomas, oligodendrogliomas, and undefined gliomas (16, 25, 26).

SDT is a novel therapy currently being investigated for the treatment of both canine and human glioma. We compared the differences in outcomes between French bulldogs treated with immunotherapy and SDT because both are novel therapies, and both therapies followed surgical HGG resection, but SDT is not a form of immunotherapy. With this therapy, dogs receive an oral dose of 5-aminolevulinic acid (5-ALA) that selectively accumulates in tumor cells, is converted into its active metabolite, protoporphyrin IX, and becomes activated by low-intensity ultrasound. Activated protoporphyrin IX generates reactive oxygen species that damage tumor cells (27). The favorable outcome of French bulldogs treated with SDT (median OS 204 days) further suggests that breed alone is not responsible for the survival disparity observed in immunotherapy-treated French bulldogs.

Collectively, these findings reveal that the poor outcome observed in the French bulldog cohort of dogs treated in our clinical trial may be unique to immunotherapy, rather than the breed’s overall response to HGG treatment in general. There are several possible reasons that French bulldog tumors do not respond as well to immunotherapy as HGG in other breeds.

First, the HGG of French bulldogs may be genetically or epigenetically different from the HGG of other breeds, particularly for genes involved in tumor-immune interactions. Overall, canine HGG is characterized by molecular heterogeneity, such that the glioma of one patient may have several key genetic distinctions from another patient (20). However, most dog breeds have very narrow genetic pools. Among 227 investigated dog breeds, there is an average inbreeding rate of 25%, with brachycephalic breeds having both significantly higher inbreeding rates and morbidity than other breeds (28). Given the rapid rise in population of French bulldogs, it seems likely that inbreeding has perpetuated the “breeding in” of HGG genes that result in an immunotherapy-resistant, breed-distinct form of HGG. Epigenetically, canine glioma can display heterogeneity in DNA methylation (21). Similar to breed-associated differences in HGG genetics, breed-associated DNA methylation profiles may also be occurring in French bulldogs compared to other breeds. Finally, although all brachycephalic breeds have similar craniofacial abnormalities, the French bulldog represents the most extreme version of brachycephaly, leading to chronic hypoxia (3). Hypoxia creates important transcriptomic changes in glioma, and may therefore contribute to the French bulldog version of HGG (29).

Poor outcomes in immunotherapy-treated French bulldogs highly suggests that if there are genetic and epigenetic differences unique to HGG-bearing French bulldogs, they are associated with local, systemic, or both local and systemic tumor-immune interactions. Breed-associated differences in immune responses have been observed in other diseases, rendering some dogs more susceptible or resistant to these conditions (30, 31). Here, inbreeding is further implicated in differences in immune function. For example, the number of dog leukocyte antigen (DLA) haplotypes varies substantially among dog breeds, with ranging from 20 to 70 haplotypes based on the genetic diversity within a given breed (32). Genetic factors can also contribute to immune responses to vaccination (33). Thus, the baseline immune function of French bulldogs may differ from that of other breeds, which may result in differences in local and systemic immune response to the tumors or to the protocols used in the immunotherapy-treated dogs. Additionally, just as the tumor genetics may differ between French bulldogs and other breeds, the immune component of the tumor microenvironment (TME) may differ as well. Clusters of differentiation (CD) molecule expression patterns are not uniform across canine glioma samples, and breed may contribute to that heterogeneity (20).

In summary, we observed that HGG-bearing French bulldogs treated specifically with vaccine and immune checkpoint-based immunotherapy had worse outcomes than similar breeds, had similar outcomes to French bulldogs treated palliatively, and had worse outcomes than French bulldogs treated with either SDT or SRT. This study provides evidence that breed-associated, treatment-resistant forms of HGG may exist among at-risk dog breeds and serves as a platform to perform studies to characterize breed-associated genetic and epigenetic features in canine HGG to further our collective understanding of this complicated disease and to investigate patient-centered therapies.

## Data Availability

The datasets analyzed for this study can be found in the Data Repository of the University of Minnesota (DRUM); https://hdl.handle.net/11299/267843.

## References

[1] Packer RMA Murphy D Farnworth MJ. Purchasing popular purebreds: investigating the influence of breed-type on the pre-purchase motivations and behaviour of dog owners. Anim Welf. (2017) 26:191–201. doi: 10.7120/09627286.26.2.191

[2] Cattanach BM Dukes-McEwan J Wotton PR Stephenson HM Hamilton RM. A pedigree-based genetic appraisal of boxer ARVC and the role of the Striatin mutation. Vet Rec. (2015) 176:492. doi: 10.1136/vr.102821, PMID: 25661582 PMC4433500

[3] O’Neill DG Packer RMA Francis P Church DB Brodbelt DC Pegram C. French bulldogs differ to other dogs in the UK in propensity for many common disorders: a VetCompass study. Canine Med Genet. (2021) 8:13. doi: 10.1186/s40575-021-00112-3, PMID: 34911586 PMC8675495

[4] Parker HG Dreger DL Rimbault M Davis BW Mullen AB Carpintero-Ramirez G. Genomic analyses reveal the influence of geographic origin, migration, and hybridization on modern dog breed development. Cell Rep. (2017) 19:697–708. doi: 10.1016/j.celrep.2017.03.079, PMID: 28445722 PMC5492993

[5] Kishimoto TE Uchida K Chambers JK Kok MK Son NV Shiga T. A retrospective survey on canine intracranial tumors between 2007 and 2017. J Vet Med Sci. (2020) 82:77–83. doi: 10.1292/jvms.19-0486, PMID: 31801930 PMC6983661

[6] José-López R Gutierrez-Quintana R de la Fuente C Manzanilla EG Suñol A Pi Castro D. Clinical features, diagnosis, and survival analysis of dogs with glioma. J Vet Intern Med. (2021) 35:1902–17. doi: 10.1111/jvim.16199, PMID: 34117807 PMC8295679

[7] Suñol A Mascort J Font C Bastante AR Pumarola M Feliu-Pascual AL. Long-term follow-up of surgical resection alone for primary intracranial rostrotentorial tumors in dogs: 29 cases (2002-2013). Open Vet J. (2017) 7:375–83. doi: 10.4314/ovj.v7i4.14, PMID: 29392117 PMC5768925

[8] Trageser E Martin T Burdekin B Hart C Leary D LaRue S. Efficacy of stereotactic radiation therapy for the treatment of confirmed or presumed canine glioma. Vet Comp Oncol. (2023) 21:578–86. doi: 10.1111/vco.12920, PMID: 37423611

[9] Debreuque M De Fornel P David I Delisle F Ducerveau MN Devauchelle P. Definitive-intent uniform megavoltage fractioned radiotherapy protocol for presumed canine intracranial gliomas: retrospective analysis of survival and prognostic factors in 38 cases (2013–2019). BMC Vet Res. (2020) 16:412. doi: 10.1186/s12917-020-02614-x, PMID: 33129320 PMC7603708

[10] Rohrer Bley C Staudinger C Bley T Marconato L Sabattini S Beckmann K. Canine presumed glial brain tumours treated with radiotherapy: is there an inferior outcome in tumours contacting the subventricular zone? Vet Comp Oncol. (2022) 20:29–37. doi: 10.1111/vco.12703, PMID: 33900018

[11] Costa RS Abelson AL Lindsey JC Wetmore LA. Postoperative regurgitation and respiratory complications in brachycephalic dogs undergoing airway surgery before and after implementation of a standardized perianesthetic protocol. J Am Vet Med Assoc. (2020) 256:899–905. doi: 10.2460/javma.256.8.899, PMID: 32223703

[12] Gruenheid M Aarnes TK McLoughlin MA Simpson EM Mathys DA Mollenkopf DF. Risk of anesthesia-related complications in brachycephalic dogs. J Am Vet Med Assoc. (2018) 253:301–6. doi: 10.2460/javma.253.3.301, PMID: 30020004

[13] Yost NM Angelastro JM. Canine Glioma as a model for human Glioblastoma. In: Glioblastoma - current evidence. IntechOpen. (2023) 11:106464. doi: 10.5772/intechopen.106464

[14] LeBlanc AK Mazcko CN. Improving human cancer therapy through the evaluation of pet dogs. Nat Rev Cancer. (2020) 20:727–42. doi: 10.1038/s41568-020-0297-3, PMID: 32934365

[15] Gómez-Oliva R Domínguez-García S Carrascal L Abalos-Martínez J Pardillo-Díaz R Verástegui C. Evolution of experimental models in the study of glioblastoma: toward finding efficient treatments. Front Oncol. (2021) 10:4295. doi: 10.3389/fonc.2020.614295, PMID: 33585240 PMC7878535

[16] Koehler JW Miller AD Miller CR Porter B Aldape K Beck J. A revised diagnostic classification of canine glioma: towards validation of the canine glioma patient as a naturally occurring preclinical model for human glioma. J Neuropathol Exp Neurol. (2018) 77:1039–54. doi: 10.1093/jnen/nly085, PMID: 30239918 PMC6181180

[17] Hansen KS Zwingenberger AL Théon AP Kent MS. Long-term survival with stereotactic radiotherapy for imaging-diagnosed pituitary tumors in dogs. Vet Radiol Ultrasound. (2019) 60:219–32. doi: 10.1111/vru.12708, PMID: 30575174

[18] Ben-Aharon O Magnezi R Leshno M Goldstein DA. Median survival or mean survival: which measure is the Most appropriate for patients, physicians, and policymakers? Oncologist. (2019) 24:1469–78. doi: 10.1634/theoncologist.2019-0175, PMID: 31320502 PMC6853128

[19] Han K Jung I. Restricted mean survival time for survival analysis: a quick guide for clinical researchers. Korean J Radiol. (2022) 23:495–9. doi: 10.3348/kjr.2022.0061, PMID: 35506526 PMC9081686

[20] Mitchell D Chintala S Fetcko K Henriquez M Tewari BN Ahmed A. Common molecular alterations in canine Oligodendroglioma and human malignant gliomas and potential novel therapeutic targets. Front Oncol. (2019) 9:780. doi: 10.3389/fonc.2019.00780 31475119 PMC6702544

[21] Amin SB Anderson KJ Boudreau CE Martinez-Ledesma E Kocakavuk E Johnson KC. Comparative molecular life history of spontaneous canine and human gliomas. Cancer Cell. (2020) 37:243–257.e7. doi: 10.1016/j.ccell.2020.01.004, PMID: 32049048 PMC7132629

[22] Brearley MJ Jeffery ND Phillips SM Dennis R. Hypofractionated radiation therapy of brain masses in dogs: a retrospective analysis of survival of 83 cases (1991–1996). J Vet Intern Med. (1999) 13:408–12. doi: 10.1111/j.1939-1676.1999.tb01454.x, PMID: 10499721

[23] Dolera M Malfassi L Bianchi C Carrara N Finesso S Marcarini S. Frameless stereotactic radiotherapy alone and combined with temozolomide for presumed canine gliomas. Vet Comp Oncol. (2018) 16:90–101. doi: 10.1111/vco.12316, PMID: 28643878

[24] Schwarz P Meier V Soukup A Drees R Besserer J Beckmann K. Comparative evaluation of a novel, moderately hypofractionated radiation protocol in 56 dogs with symptomatic intracranial neoplasia. J Vet Intern Med. (2018) 32:2013–20. doi: 10.1111/jvim.15324, PMID: 30308086 PMC6272041

[25] Diangelo L Cohen-Gadol A Heng HG Miller MA Hague DW Rossmeisl JH. Glioma mimics: magnetic resonance imaging characteristics of granulomas in dogs. Front Vet Sci. (2019) 6:286. doi: 10.3389/fvets.2019.00286 31555671 PMC6722480

[26] Amphimaque B Durand A Oevermann A Vidondo B Schweizer D. Grading of oligodendroglioma in dogs based on magnetic resonance imaging. J Vet Intern Med. (2022) 36:2104–12. doi: 10.1111/jvim.16519, PMID: 36366870 PMC9708455

[27] Raspagliesi L D’Ammando A Gionso M Sheybani ND Lopes MB Moore D. Intracranial Sonodynamic therapy with 5-Aminolevulinic acid and sodium fluorescein: safety study in a porcine model. Front Oncol. (2021) 11:679989. doi: 10.3389/fonc.2021.679989, PMID: 34235081 PMC8256685

[28] Bannasch D Famula T Donner J Anderson H Honkanen L Batcher K. The effect of inbreeding, body size and morphology on health in dog breeds. Canine Med Genet. (2021) 8:12. doi: 10.1186/s40575-021-00111-4, PMID: 34852838 PMC8638537

[29] Marallano VJ Ughetta ME Tejero R Nanda S Ramalingam R Stalbow L. Hypoxia drives shared and distinct transcriptomic changes in two invasive glioma stem cell lines. Sci Rep. (2024) 14:7246. doi: 10.1038/s41598-024-56102-5, PMID: 38538643 PMC10973515

[30] Álvarez L Marín-García PJ Llobat L. Genetic haplotypes associated with immune response to Leishmania infantum infection in dogs. Vet Res Commun. (2023) 47:1675–85. doi: 10.1007/s11259-023-10123-z, PMID: 37059873

[31] Woolhead VL Szladovits B Chan A Swann JW Glanemann B. Breed predispositions, clinical findings, and prognostic factors for death in dogs with nonregenerative immune-mediated anemia. J Vet Intern Med. (2021) 35:252–60. doi: 10.1111/jvim.15986, PMID: 33617109 PMC7848385

[32] Dreger DL Rimbault M Davis BW Bhatnagar A Parker HG Ostrander EA. Whole-genome sequence, SNP chips and pedigree structure: building demographic profiles in domestic dog breeds to optimize genetic-trait mapping. Dis Model Mech. (2016) 9:1445–60. doi: 10.1242/dmm.027037, PMID: 27874836 PMC5200897

[33] Blake JM Thompson J HogenEsch H Ekenstedt KJ. Heritability and genome-wide association study of vaccine-induced immune response in beagles: a pilot study. Vaccine. (2024) 42:3099–106. doi: 10.1016/j.vaccine.2024.03.076, PMID: 38604911 PMC11144447

