# Supplementary material for: Immunotherapy yields breed-specific worst survival outcomes among three investigated therapies in French bulldogs with high-grade glioma
Source: Front Vet Sci. 2025 Mar 19;12:1532439. doi: 10.3389/fvets.2025.1532439 (PMC11961989; doi:10.3389/fvets.2025.1532439)
Supplement: Supplementary file 1 [file Table_1.docx]

Supplementary file 1: Immunotherapy treatment protocols

Autologous tumor lysate and OX40L therapy:

Autologous tumor lysate vaccines were prepared by mincing fresh tumor samples and culturing single-cell suspensions at 37°C in 5% O^2^ (15). Multiple freeze-thaw cycles were used to lyse cultured tumor cells, which were irradiated at 20Gy. Immunotherapy protocols consisted intradermal injection of the autologous tumor lysate (~ 500 µg/vaccine) after topical application of imiquimod (1 packet 5% cream/12.5 g), a Toll-like receptor 7 agonist, as an immune adjuvant. OX40L (0.5 mg/injection if 5-20 kg BW, 1 mg/injection if 20-35 kg BW), a co-stimulatory molecule for T cell activation, was given as intradermal injections along with the lysate vaccines, and without lysate for 4 more days following the initial injection. Starting 10-14 days after maximal, safe tumor resection, the dogs received the autologous tumor lysate and OX40L once weekly for three weeks, then every 4 weeks for three treatments. Some dogs also received adjuvant therapy with temozolomide (150 mg/m^2^ x BSA/1.73 m^2^, PO, qD) for 5 days every 28-day cycle for 5 cycles. All dogs underwent recheck MRIs at 3, 6 and 12 months after the immediate postoperative MRI. Progression-free survival (PFS) was recorded as the time from tumor resection to tumor recurrence on MRI and overall survival (OS) was recorded as time from tumor resection to patient death from euthanasia or natural causes.

Combination gene therapy:

After maximal, safe tumor resection, combination gene therapy with high-capacity adenoviral (HC-Ad) Tetracycline On (TetOn) Fms-related tyrosine kinase 3 ligand (Flt3L)and HC-Ad thymidine kinase (TK) was administered by 15 (100-µl) injections into the brain parenchyma surrounding the resection cavity. After surgery, ganciclovir (10 mg/kg, PO, BID), the prodrug for TK, was given for ten days and doxycycline (5 mg/kg, PO, BID), to activate expression of Flt3L, was given for 4 weeks. In addition, each dog received adjuvant chemotherapy with low-dose temozolomide (50 mg/m^2^ x BSA/1.73 m^2^, PO, qD) for 14 days then 4 weeks later cyclic high-dose temozolomide (150 mg/m^2^ x BSA/1.73 m^2^, PO, qD) for 5 days every 28-day cycle for 5 cycles. All dogs underwent recheck MRIs at 2, 6 and 12 months after the immediate postoperative MRI. Progression-free survival (PFS) was recorded as the time from tumor resection to tumor recurrence on MRI and overall survival (OS) was recorded as time from tumor resection to patient death from euthanasia or natural causes.

Autologous tumor lysate and CD200 immune checkpoint inhibition therapy:

Autologous tumor lysate vaccines were prepared by mincing fresh tumor samples and culturing single-cell suspensions at 37°C in 5% O^2^ (15). Multiple freeze-thaw cycles were used to lyse cultured tumor cells, which were irradiated at 20Gy. Immunotherapy protocols consisted intradermal injection of the autologous tumor lysate (~ 500 µg protein/vaccine), and CD200AR-L (10 μg/kg), a peptide ICI, after topical application of imiquimod (1 packet 5% cream/12.5 g), a Toll-like receptor 7 agonist, as an immune adjuvant. Starting 10-14 days after maximal, safe tumor resection, the dogs received the autologous tumor lysate, adjuvant, and CD200AR-L once weekly for three weeks, then every 4 weeks for three treatments, then every seven (± 1) weeks until tumor progression, patient death, or withdrawal from the study. All dogs underwent recheck MRIs every four months. Progression-free survival (PFS) was recorded as the time from tumor resection to tumor recurrence on MRI and overall survival (OS) was recorded as time from tumor resection to patient death from euthanasia or natural causes.
